# Influence of Low pH on the Microhardness and Roughness Surface of Dental Composite—A Preliminary Study

**DOI:** 10.3390/ma17143443

**Published:** 2024-07-12

**Authors:** Leszek Szalewski, Dorota Wójcik, Monika Sowa, Vladyslav Vivcharenko, Krzysztof Pałka

**Affiliations:** 1 Digital Dentistry Lab, Department of Dental and Maxillofacial Radiodiagnostics, Medical University of Lublin, 20-059 Lublin, Poland; 2 Department of Dental Prosthetics, Medical University of Lublin, 20-059 Lublin, Poland; dorota.wojcik@umlub.pl; 3 Deptartment of Materials Engineering, Lublin University of Technology, 20-618 Lublin, Poland; d577@pollub.edu.pl (M.S.); k.palka@pollub.pl (K.P.); 4 Department of Tissue Engineering & Regenerative Medicine, Medical University of Lublin, 20-059 Lublin, Poland

**Keywords:** restorative dentistry, aesthetic dentistry, biomechanics, hardness, microscopy, nano-filled composite, surface degradation

## Abstract

Dental composites are gaining great popularity in restorative dentistry because of their aesthetic appeal and capacity to replicate the natural color of teeth. Nevertheless, their lifespan and durability rely on various factors, such as the polishing technique and the environmental conditions they are exposed to. The study aimed to assess the influence of the method of final polishing of dental composite on the surface roughness and microhardness of materials also considering the environment of different pHs. Disc-shaped samples (5 mm diameter and 2 mm thickness) have been prepared for microhardness and roughness tests from two dental composites: A2 Clearfil Majesty ES 2 Classic and A2D Clearfil Majesty Premium. One-third of samples were polished with polishing discs, OptiDisc, another one-third of samples were polished with Eve Diacomp Twist rubbers and polishing brush with diamond particles, and rest of the samples were stored without any polishing (the control group). Tested materials were incubated in distilled water or acidic buffer (pH = 2) for 3 weeks at a temperature of 37 °C. No statistically significant differences were found for roughness for the two materials tested after incubation in liquids. A decrease in Vicker microhardness was found for Clearfil Majesty ES 2 Classic after soaking in a low pH liquid, and no such relationship was found for Clearfil Majesty Premium. The improved resistance of these materials to the negative oral environment may result in the longer survival of composite restorations in patients with poor diet or diseases, causing a decrease in oral pH.

## 1. Introduction

Dental composites have become increasingly popular in restorative dentistry due to their aesthetic appeal and ability to mimic the natural color of teeth. However, their longevity and durability depend on a range of aspects, including the method of polishing and the environmental conditions they are exposed to [1,2]. The polishing of dental composites is crucial to achieve a smooth and glossy surface, which ensures the proper functioning of the stomatognathic system, reduces the accumulation of plaque, and prevents staining [3]. However, polishing can also affect the surface roughness and, subsequently, the mechanical and chemical properties of restoration [4]. Moreover, exposure to acidic environments can cause the degradation of the resin matrix, leading to the deterioration of mechanical properties, discoloration, and microleakage [5]. Recently, several studies have investigated the effect of different polishing methods and acidic environments on the properties of dental composites [6,7,8,9,10]. These studies have used various techniques to evaluate the surface roughness, microhardness, color stability, and degree of conversion of the composites. Composite materials can be polished with abrasive discs, rubber bands, and brushes, with the addition of polishing pastes. In addition, all these methods can be combined in different configurations. Erosion factors from the diet are also not indifferent to the surface of fillings made of composite materials. Low pH values usually have a negative effect on the surface of the fillings [11,12]. Various test liquids were used in the tests: chemical solutions with a known pH or dietary ingredients— drinks (e.g., Cola, tea, orange juice, etc.).

One of the challenges in comparing the results of these studies is the lack of standardization of the testing protocols, including the polishing methods, the type and duration of exposure to acidic environments, and the techniques used to evaluate the properties of the composites. Differences in the results of different studies may be due to diverse sample preparation methodologies. As demonstrated by Ko et al. in their paper on light beam in composite polymerization tips, composite materials undergo inhomogeneous polymerization during exposure [13]. Therefore, it is extremely important in research work to test a similar surface of each specimen with the indenter of a hardness tester, as the results in the center and on the sides of the same specimen can differ significantly. Also, an increase in the distance between the polymerization lamp tip and the composite has an effect on the mechanical properties of the composite materials (such as microhardness) as shown in their study by Hasanain et al. [14]. Additionally, the elimination of the oxygen inhibition layer as well as the polishing of the composite materials can affect the obtained Vickers microhardness results, as shown in their study by Carrillo-Marcos et al. [15]. Both polishing and elimination of the oxygen inhibition layer result in an increase in Vickers microhardness values and a decrease in surface roughness.

Therefore, it is essential to establish standardized testing protocols to enable reliable and accurate comparisons of the properties of dental composite resins.

The null hypothesis of the current study was that the type of polishing and the incubation environment had no effect on microhardness and surface roughness.

## 2. Materials and Methods

### 2.1. Specimens’ Preparation

Forty-eight disc-shaped samples (5 mm diameter and 2 mm thickness) have been prepared for microhardness and roughness tests for each material. Specimens were produced using a stainless steel mold placed on a microscope slide to achieve a flat surface. The following nanohybrid composites were used in tests, all of the A2 shade: Clearfil Majesty ES 2 Classic (A2C) (Kuraray Europe GmbH, Hattersheim am Main, Germany) and Clearfil Majesty Premium (A2D) (Kuraray Europe GmbH, Hattersheim am Main, Germany). The choice of materials was dictated by differences in application. During filling corrections, the dentin material (A2D) is sometimes exposed beneath the enamel layer (A2C). It is important to check that the dentin material has the same low pH resistance properties as the enamel layer. Advantageously, both materials have a very similar composition: organic matrix—Bis-GMA, dimethacrylate, and photoinitiator (camphorquinone); inorganic filler (78 wt.%)—silanizated barium glass filler, and pre-polymerized organic filler.

Samples were polymerized using a high-powered LED LCU (Mini LED III Supercharged, Acteon Group, Merignac, France). Samples were cured using 10 s pulse-cure mode (total energy = 20 J/cm^2^) without any oxygen inhibition layer protection. After photopolymerization, the specimens were released from the mold. The steel mold blocked the light from the side of the samples, which made it possible to obtain reproducible results. There was no risk of false results due to the lateral polymerization of the sample. Next, the specimens were examined under magnification (3.5×) for the presence of air bubbles, and defective specimens were excluded from the study. All specimens were sandblasted (AirNGo, Acteon Group, Merignac, France) with prophylaxis sand (KaVo PROPHYPearls, KaVo Dental GmbH, Warthausen, Germany) for 10 s to eliminate the oxygen inhibition layer.

### 2.2. Study Group

Two additional groups of samples (N = 8 each) were prepared for polishing and conditioning experiments. One group was polished using a series of OptiDisc polishing discs (KerrDental, Brea, USA) from coarse to medium, fine, and extra-fine grits. The other group was polished using a 2-step polishing system with Eve Diacomp Twist rubbers (EVE Ernst Vetter GmbH, Birkenfeld, Germany) followed by polishing with a brush containing diamond particles (EVE Ernst Vetter GmbH, Germany). The final group served as a control and was left unpolished.

**Figure 1:**
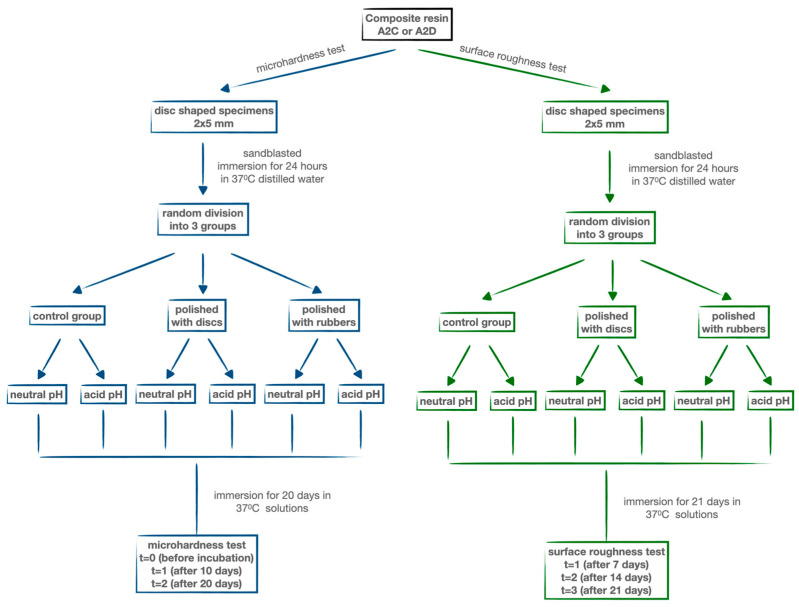
Flow chart of the research process.

After polishing, all specimen groups were immersed in distilled water at 37 °C for 24 h. Subsequently, each of the three main groups was further divided into two subgroups. One subgroup was incubated in 100 mL of distilled water [16], while the other was incubated in 100 mL of acidic solution (pH = 2) (Alfachem Sp. z o.o., Lublin, Poland) for 3 weeks at 37 °C (Figure 1).

The conditioning time in the acidic solution was determined based on guidelines from the study by Gawriołek et al. [17]. According to this protocol, one cup of coffee (150 mL) is assumed to have a 1 min contact time with the oral cavity. Considering a daily consumption of 2 cups (300 mL), each 7 days of sample storage can be correlated to 2 years of material conditioning in the oral environment.

### 2.3. Vickers Microhardness Test

Microhardness measurements were performed using the Vickers method on an FM-800 hardness tester (Future-Tech, Kawasaki-City, Japan) with a load of 0.4905 N, as described in the study by Szalewski et al. [16]. Ten measurements were taken on the photopolymerization side of each sample. The measurements were conducted at three time points: T0 (before incubation), T1 (after 10 days of incubation in the test solution), and T2 (after 20 days of incubation).

### 2.4. Roughness Test

Surface roughness of the specimens was assessed using an Olympus LEXT OLS5100 3D laser scanning microscope (Olympus Corporation, Tokyo, Japan). Due to the directional nature of the machined surfaces, roughness measurements were conducted using a surface method with the laser microscope. Consistent with the microhardness measurements, the same photopolymerization side of each sample was utilized for these tests. The Sa parameter (arithmetical mean height) was measured four times per sample. Measurements were taken at three time points: T1 (after 7 days of incubation in the test solution), T2 (after 14 days), and T3 (after 21 days).

### 2.5. Scanning Electron Microscopy (SEM)

The surface morphology of the samples was examined using scanning electron microscopy (SEM). SEM observations were conducted with a Nova NanoSEM 450 (FEI, Eindhoven, The Netherlands) operating in the low-vacuum mode with an accelerating voltage of 5 kV.

### 2.6. Statistical Methods

Hypotheses related to the differences between the mean values of the Vickers microhardness obtained for incubation solution types and the time of incubation and the polishing protocol were verified by mixed-model ANOVA assumptions analysis, followed by mixed-model ANOVA and post hoc tests. The hypotheses were verified using the significance level of *p* = 0.05. Mean values and standard deviations were calculated. In the first step, the normality of the distribution was analyzed using the Shapiro–Wilk’s test, along with the analysis of skewness and kurtosis. In addition, Levene’s test was included in the analyses to assess the homogeneity of the variance.

Hypotheses related to the differences between the mean values of the roughness test obtained for incubation solution types and the time of incubation were verified by mixed-model ANOVA assumptions analysis, followed by mixed-model ANOVA and post hoc tests. The hypotheses were verified using the significance level of *p* = 0.05. Mean values and standard deviations were calculated. In the first step, the normality of the distribution was analyzed using the Shapiro–Wilk test, along with the analysis of skewness and kurtosis. In addition, Levene’s test was included in the analyses to assess the homogeneity of the variance. The analyses were conducted using the IBM SPSS Statistics 28 software (International Business Machines Corporation, New York, NY, USA).

## 3. Results

### 3.1. Vickers Microhardness Test

**Figure 2:**
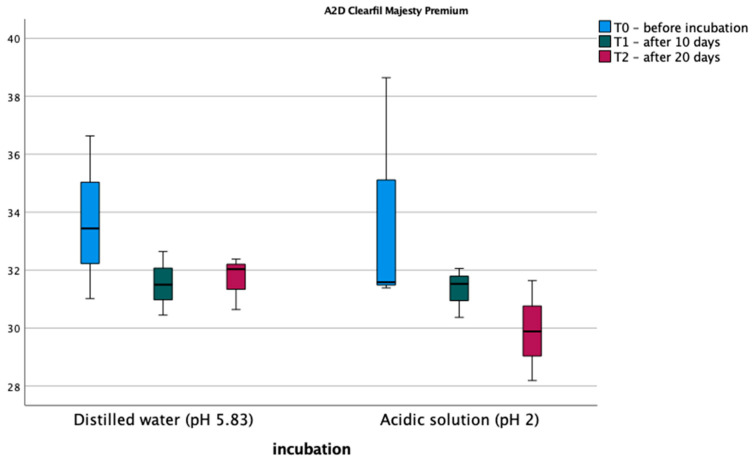
Average Vickers microhardness values for A2D material.

**Figure 3:**
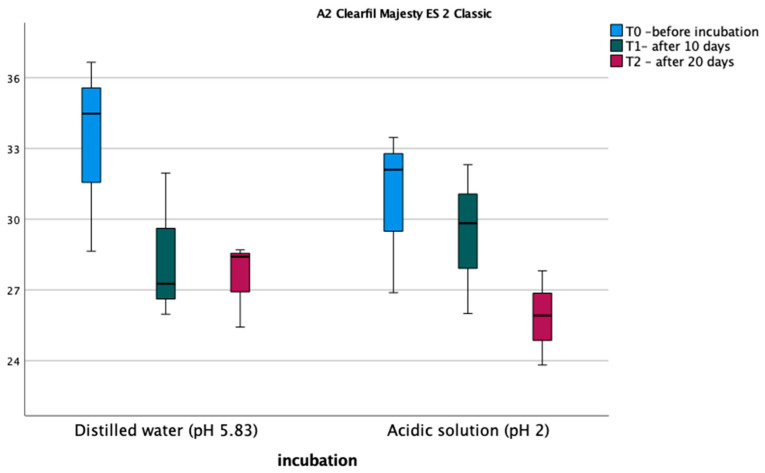
Average Vickers microhardness values for A2C material.

The results of the microhardness measurements are shown in Figure 2 and Figure 3. Normality tests of the distribution indicate that the normality of the distribution is met in each measurement and soaking method. The assumption of sphericity was met (Mauchly’s W = 0.604, *p* = 0.469). An analysis of variance was performed in a mixed scheme, where the within-subject factors were Vickers microhardness before soaking, after 10 days, and after 20 days of induction, the between-subject variables, and the type of fluid in which the samples were soaked. The analysis did not detect significant differences between measurements for the A2D composite; on the other hand, for the A2C composite, significant differences were found between measurements, and the main effect of measurement was statistically significant, F (2,8) = 10.778, *p* = 0.005, η^2^ = 0.729. Bonferroni post hoc tests did not show statistically significant differences between measurements for the two materials. Also, the type of polishing had no statistically significant effect on the Vickers microhardness of the composite before and after incubation in the different pH solutions.

### 3.2. Roughness Test

**Figure 4:**
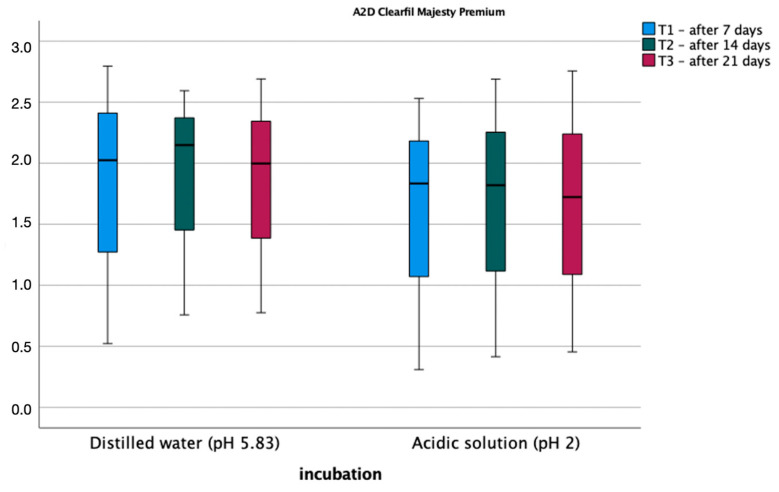
Average roughness values for A2D material.

**Figure 5:**
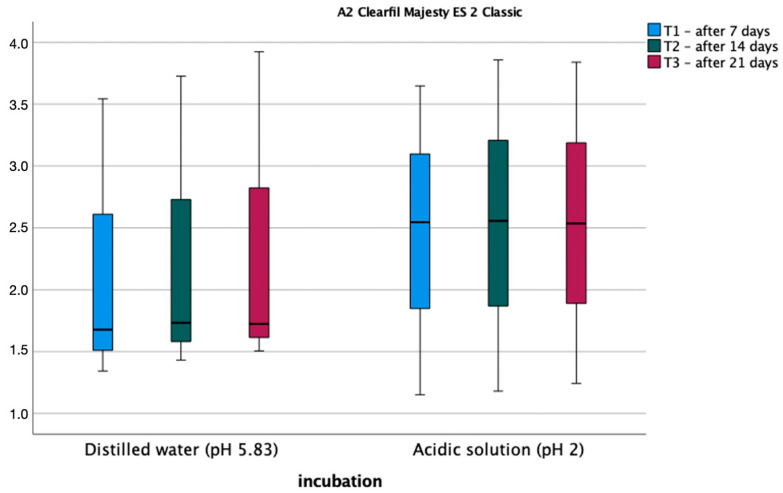
Average roughness values for A2C material.

Figure 4 and Figure 5 show the average roughness values for the tested materials. The assumption of sphericity was met (Mauchly’s W = 0.716, *p* = 0.606). An analysis of variance was performed in a mixed design scheme, where the within-subject factors were roughness after 1 week, 2 weeks, and 3 weeks of soaking, the between-subject variables, and the type of fluid in which the sample was soaked. Bonferroni post hoc tests for both materials showed no statistically significant differences between measurements. Also, the type of polishing had no statistically significant effect on the roughness of the composite before and after incubation in the different pH solutions.

### 3.3. Scanning Electron Microscopy (SEM)

**Figure 6:**
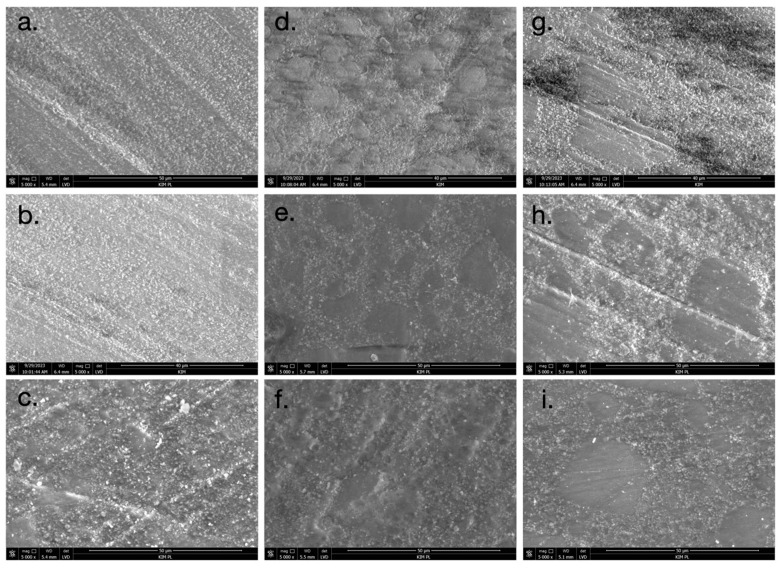
SEM images of A2C composite (magnification: ×5000): (**a**) A2C t = 0; (**b**) A2C t = 21 days, pH neutral; (**c**) A2C t = 21 days, pH acidic; (**d**) A2C_G t = 0; (**e**) A2C_G t = 21 days, pH neutral; (**f**) A2C_G t = 21 days, pH acidic; (**g**) A2C_K t = 0; (**h**) A2C_K t = 21 days, pH neutral; and (**i**) A2C_K t = 21 days, pH acidic.

**Figure 7:**
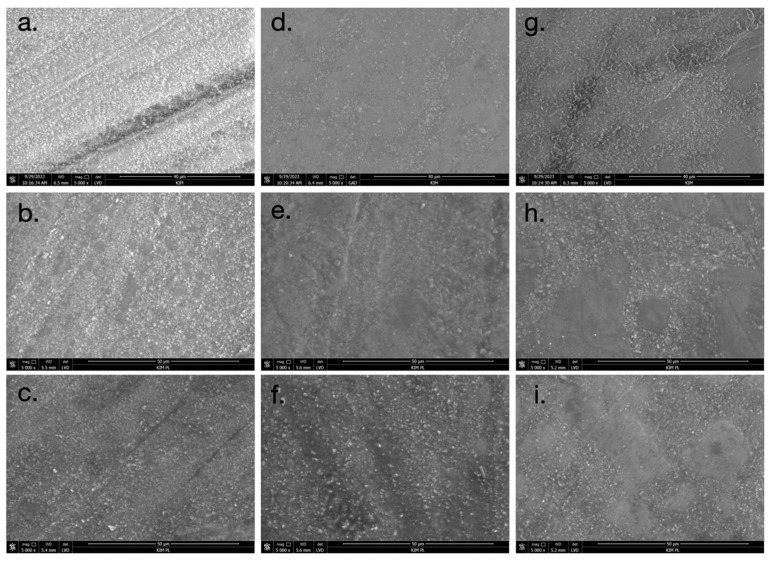
SEM images of A2D composite (magnification: ×5000): (**a**) A2D t = 0; (**b**) A2D t = 21 days, pH neutral; (**c**) A2D t = 21 days, pH acidic; (**d**) A2D_G t = 0; (**e**) A2D_G t = 21 days, pH neutral; (**f**) A2D_G t = 21 days, pH acidic; (**g**) A2D_K t = 0; (**h**) A2D_K t = 21 days, pH neutral; and (**i**) A2D_K t = 21 days, pH acidic.

The SEM images presented in Figure 6 and Figure 7 reveal distinct differences in the surface morphology of the composite resin materials. The surfaces of the untreated, unpolished specimens exhibited the smoothest appearance (Figure 6a and Figure 7a). When polishing rubbers were used, the SEM images showed evidence of composite resin filler loss on the surface (Figure 6d and Figure 7d). This suggests that the polishing process with rubbers resulted in the removal of some of the resin filler particles. In the case of abrasive discs, the directionality of the polishing process is visible on the surface (Figure 6g and Figure 7g). This is likely due to the standardized unidirectional polishing protocol used to maintain consistency across the samples. After soaking the specimens in both neutral pH and acidic pH solutions, the degradation of the matte surface was observed (Figure 6e,f,h,i and Figure 7e,f,h,i). The changes were more pronounced in the acidic pH conditions, where eroded areas of the surface could be seen. Importantly, no cracks or mechanical damage were detected on the surfaces of the samples during the polishing procedures. This indicates that the polishing protocols employed did not result in any visible structural damage to the composite resin materials.

## 4. Discussion

The null hypothesis was confirmed in the case of the A2D material for both tested parameters, and in the case of the A2C material, the roughness is not affected by the tested factors, but the Vickers microhardness of the material decreases after a 3-week incubation at low pH.

A decrease in oral pH can be attributed to various factors, including dietary abnormalities, impaired mineral balance, or systemic diseases. This acidic environment can adversely affect the mechanical and functional properties of dental restorative materials. The maintenance of excellent mechanical properties and resistance to environmental changes in the oral cavity is crucial for composite materials used in dentistry. Over the years, manufacturers have refined both the application and polymerization procedures of composite materials, as well as their composition, to achieve optimal properties for dental restorations. In a study by Kumari et al., none of the tested composite materials exhibited changes in Vickers microhardness after exposure to citric acid (low pH) compared to storage in neutral pH (air) [18]. Our research corroborates these findings for the A2D material; however, the A2C material demonstrated a decrease in Vickers microhardness. Hamdy et al. similarly reported no statistically significant difference in the Vickers microhardness of tested composite materials after immersion in mouthwashes [19], which partially aligns with our results. It should be noted, however, that their methodology involved only a 24 h immersion period, which may be insufficient to observe significant changes. Kumari et al.’s study also revealed varying initial microhardness values among the tested materials, ranging from 28.35 to 96.76 HV. Contrasting results were obtained by Barve et al., who observed a statistically significant reduction in Vickers microhardness values for Filtek Z250 material stored in Cola (low pH) after a 15-day immersion period compared to the control sample (distilled water) [20]. Unal et al. also noted a decrease in the Vickers microhardness of composite materials after 14 days of exposure to gastric acid, simulating conditions associated with diseases causing frequent vomiting, such as bulimia [21]. In their study, the extent of Vickers microhardness reduction varied depending on the material tested, ranging from 35% (Beautifil II) to 16% (Filtek Z550). Similarly, Vecek et al. reported a reduction in the Vickers microhardness of composite materials by 8–28% after a 30-day incubation in a green smoothie, which aligns with our findings for the A2C material [22]. Additionally, composite materials in the oral cavity are exposed to other factors, such as tooth-whitening products. Fernandes et al. demonstrated that tooth-whitening formulations based on 35% hydrogen peroxide or 16% carbamide peroxide can cause up to a 10% decrease in Knoop microhardness [23].

The effects of low pH environments on the surface roughness of dental composite materials have been examined in several studies, yielding mixed results. Kumari et al. observed that exposure to a low pH citric acid solution led to a statistically significant decrease in surface roughness for half of the tested composite materials (Tetric Evo Ceram and Filtek Z350), while the remaining materials (Clearfil Majesty and EverX) showed no change in roughness [18]. In contrast, Camilotti et al. reported an increase in roughness after just 30 days of immersion in low pH solutions (pH = 2.73, 2.74, 3.58), with further increases noted over extended periods of 90 and 180 days [24]. Tavangara et al. conducted a study on three different composite materials polished with Soflex discs and found that soaking in low pH liquids (pH = 2.47 for Coke and pH = 5.41 for coffee) resulted in an increase in roughness compared to a control group soaked in distilled water [25]. These findings diverge from those of the current study, which found no statistically significant difference in surface roughness after soaking the composite materials in an acidic liquid. The research by Vaidya et al. demonstrated that liquid composite materials exhibit increased roughness when exposed to acidic beverages, including energy drinks and alcoholic beverages [26]. However, bulk-fill composite materials showed resilience to short-term acid exposure, with no significant roughness changes observed after a 60 min immersion combined with brushing simulation [10]. This suggests that newer composite materials may possess enhanced acid resistance, although mechanical hygiene practices continue to significantly influence surface characteristics. Lepri and Palma-Dibb’s study further nuanced these findings, indicating that acid immersion alone may not significantly alter roughness compared to neutral pH conditions [27]. However, the combination of acid exposure and brushing resulted in statistically significant increases in roughness. These results underscore the complex interplay between environmental factors and mechanical stress in determining the surface properties of dental composites. The divergent outcomes across studies highlight the need for standardized methodologies and consideration of material-specific responses when evaluating the effects of acidic environments on dental composites.

The roughness of composite materials significantly influences their longevity in the patient’s mouth. Guo et al. demonstrated that materials with greater initial roughness tend to wear out more quickly [8]. The process is further accelerated when these materials are soaked in low pH fluids (pH = 5.5) compared to a control group (pH = 7.05). This acceleration may be attributed to the loss of ceramic particles during polishing, as observed in SEM studies. Our study also noted a clear loss of filler particles post-polishing with rubbers or polishing discs, an effect that intensifies after exposure to acidic pH. The literature reports similar findings, indicating an increase in roughness following the finishing of composite materials with rotary tools [7]. Notably, composites treated with Enchance rubbers exhibited higher surface roughness compared to those polished with Soflex discs similar to our results. SEM images from our study revealed the dissolution of the organic matrix in composite materials after aging in both neutral and acidic solutions. Consequently, composites with a higher filler content are more resistant to low pH environments. This observation is corroborated by Sideridou et al., who reported that the degradation of composite surfaces occurs due to the hydrolysis of the organic matrix and the release of filler particles [28]. This degradation leads to a decrease in Vickers microhardness and an increase in material roughness.

Based on the obtained results, it can be concluded that the proper finishing of composite materials yields surfaces with adequate smoothness, which remain largely unaffected by low pH dietary conditions. Nevertheless, patients should be advised that the consumption of acidic foods may lead to material degradation, manifesting as decreased Vickers microhardness. This degradation could potentially result in compromised marginal integrity, discoloration, cracking, and ultimately necessitate restoration replacement. To enhance the mechanical properties of composite restorations, such as Vickers microhardness and flexural strength, dental practitioners should consider employing extended polymerization times for the final layer or utilizing polymerization units with higher energy output [29,30]. These measures may contribute to improved longevity and performance of composite restorations in the oral environment.

## 5. Conclusions

The study found a decrease in Vickers microhardness for one test material (Clearfil Majesty Es-2) after immersion in a low pH liquid (pH = 2) compared to a neutral solution (pH = 5.83). However, there was no significant correlation between the soaked fluid and the roughness of the composite materials. These results may suggest an improvement in the mechanical properties of the new composite materials, as exemplified by Clearfil Majesty Es-2 and Clearfil Majesty Premium. The enhanced resistance of these materials to the hostile oral environment may lead to longer survival of composite restorations in patients with poor dietary habits or diseases, causing a decrease in oral pH.

## Data Availability

Data are contained within the article.
